# Shot in the Heart

**DOI:** 10.5811/westjem.2015.11.28464

**Published:** 2016-01-12

**Authors:** Abdullah Bakhsh, Bryan Morse, Christopher Funderburk, Patrick Meloy, Katie Dean, Jeffrey Siegelman, Todd Taylor

**Affiliations:** * Emory University School of Medicine, Department of Emergency Medicine, Atlanta, Georgia; † Emory University School of Medicine, Department of Surgery, Atlanta, Georgia

A 25-year-old male was brought in by ambulance to the emergency department (ED) after sustaining a gunshot wound to his chin and left shoulder. Upon arrival to the ED, his airway was intact without evidence of blood in the oropharynx. He was found to have slightly diminished breath sounds on the left side, with respirations at 34 breaths per minute, a blood pressure of 72/50mmHg, and a heart rate of 76 beats per minute with cool extremities and poor peripheral pulses. His focused abdominal sonography in trauma exam showed a foreign body within the right ventricle without a pericardial effusion (Figure 1 and Video). An upright portable chest radiograph performed immediately thereafter showed blunting of the left costophrenic angle with a bullet fragment overlying the cardiac shadow (Figure 2).

## Diagnosis: Traumatic Ventricular Septal Defect

He was taken emergently to the operating room (OR) for exploration where he was found to have a ventricular septal defect (VSD), which was repaired. After a prolonged hospital stay, he was discharged to a long-term acute care facility for ventilator weaning. This patient is no longer dependent on the ventilator and is neurologically intact with a Glasgow Coma Scale of 15 and a Modified Rankin Scale of zero. He currently lives with a retained bullet fragment in his right ventricle. Traumatic VSD is a rare occurrence in cases of penetrating cardiac injury, with an incidence of only 1% to 5%.^1^ In patients who respond to resuscitation, as evidenced by systolic blood pressure >60mmHg to <100mmHg, there is evidence that they would benefit from emergency thoracotomy (ET [immediate thoracotomy performed in the OR]), as opposed to ED thoracotomy (EDT [immediate thoracotomy performed in the ED]). In these patients, survival can reach 75% to hospital discharge. Those patients who do not respond to the initial resuscitative effort have only a 25% survival rate after an EDT.^2^ Ultrasonography, performed during the initial survey of a trauma patient, has promise as a diagnostic modality for a variety of foreign bodies with a sensitivity and specificity of 96.7% and 70%, respectively.^3^ The chest film in the trauma bay showed a bullet fragment overlaying the cardiac shadow; however, given that only a single anteroposterior view was obtained it was difficult to ascertain the exact location of the bullet fragment. The use of ultrasound has the ability to detect intracardiac foreign bodies.

## Figures and Tables

**Figure 1:**
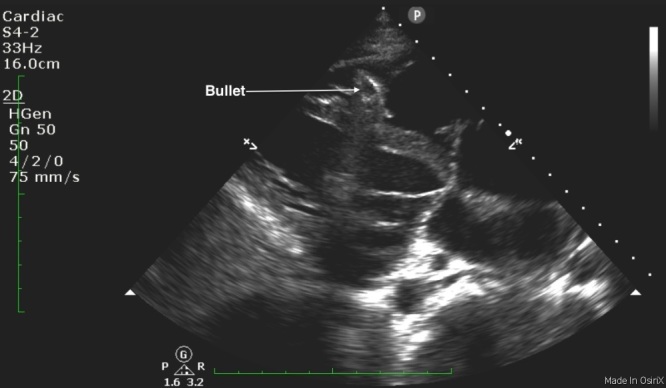
Parasternal long axis view demonstrating a bullet fragment within the right ventricle.

**Figure 2:**
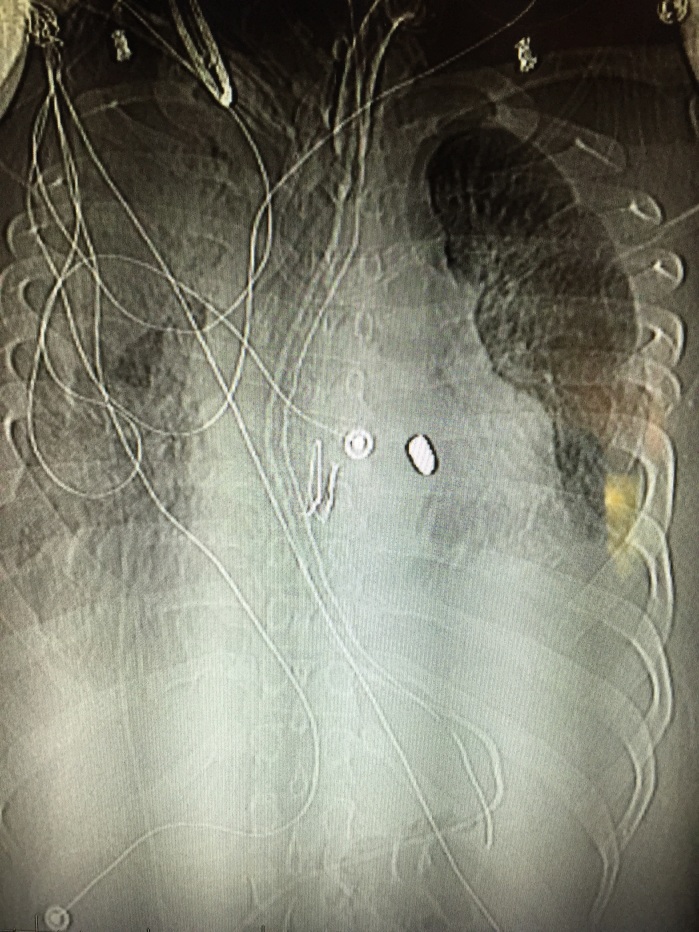
Anteroposteior chest x-ray demonstrating bullet fragment overlying the cardiac shadow.

**Video:** Parasternal long axis clip demonstrating a bullet fragment within the right ventricle.
